# Supplementary material for: The in-tissue molecular architecture of β-amyloid pathology in the mammalian brain
Source: Nat Commun. 2023 May 17;14:2833. doi: 10.1038/s41467-023-38495-5 (PMC10192217; doi:10.1038/s41467-023-38495-5)
Supplement: Supplementary file 3 — Description of additional supplementary files [file 41467_2023_38495_MOESM3_ESM.pdf]

<u>Description of additional supplementary files</u>

Supplementary Data 1 : Statistics and constituents of in-tissue tomograms from MX04-labelled amyloid plaques.

Supplementary Data 2 : Statistics and constituents of in-tissue tomograms from control (*App*$_{WT/WT}$ – *Psd95*$^{GFP/GFP}$) mice.

Supplementary Movie 1 : Video showing tomographic volume at central region of MX04-labelled amyloid plaque. Related to **Fig. 2a**.

Supplementary Movie 2 : Video showing tomographic volume at central region of MX04-labelled amyloid plaque. Related to **Fig. 2b**.

Supplementary Movie 3 : Video showing tomographic volume at the peripheral region of MX04-labelled amyloid plaque. Related to **Fig.** 3a.

Supplementary Movie 4 : Video showing tomographic volume at the peripheral region of MX04-labelled amyloid plaque. Related to **Fig. 3a**.

Supplementary Movie 5 : Video showing tomographic volume of ex vivo purified amyloid. Related to **Supplementary Fig. 9a.**
