# Supplementary material for: The in-tissue molecular architecture of β-amyloid pathology in the mammalian brain
Source: Nat Commun. 2023 May 17;14:2833. doi: 10.1038/s41467-023-38495-5 (PMC10192217; doi:10.1038/s41467-023-38495-5)
Supplement: Supplementary file 11 — Reporting Summary [file 41467_2023_38495_MOESM11_ESM.pdf]

nature portfolio

Corresponding author(s): Dr René Frank, Professor Neil Ranson, Professor Sheena Radford

Last updated by author(s): 16 Nov 2022

Reporting Summary

Nature Portfolio wishes to improve the reproducibility of the work that we publish. This form provides structure for consistency and transparency in reporting. For further information on Nature Portfolio policies, see our Editorial Policies and the Editorial Policy Checklist.

Statistics

For all statistical analyses, confirm that the following items are present in the figure legend, table legend, main text, or Methods section.

n/a	Confirmed	
☐	☒	The exact sample size (*n*) for each experimental group/condition, given as a discrete number and unit of measurement
☐	☒	A statement on whether measurements were taken from distinct samples or whether the same sample was measured repeatedly
☐	☒	The statistical test(s) used AND whether they are one- or two-sided<br>*Only common tests should be described solely by name; describe more complex techniques in the Methods section.*
☒	☐	A description of all covariates tested
☒	☐	A description of any assumptions or corrections, such as tests of normality and adjustment for multiple comparisons
☐	☒	A full description of the statistical parameters including central tendency (e.g. means) or other basic estimates (e.g. regression coefficient) AND variation (e.g. standard deviation) or associated estimates of uncertainty (e.g. confidence intervals)
☐	☒	For null hypothesis testing, the test statistic (e.g. *F*, *t*, *r*) with confidence intervals, effect sizes, degrees of freedom and *P* value noted<br>*Give P values as exact values whenever suitable.*
☒	☐	For Bayesian analysis, information on the choice of priors and Markov chain Monte Carlo settings
☒	☐	For hierarchical and complex designs, identification of the appropriate level for tests and full reporting of outcomes
☒	☐	Estimates of effect sizes (e.g. Cohen's *d*, Pearson's *r*), indicating how they were calculated

Our web collection on statistics for biologists contains articles on many of the points above.

Software and code

Policy information about availability of computer code

Data collection	SerialEM 3.5.9, EPU-3.0
Data analysis	Imod 4.10.30, Matlab2018b, Cryolo 1.6.1, Chimera1.13.1, ChimeraX1.1.<br>CryoET data processing (all available and referenced in methods): Gctf1.18-b2, IMOD 4.10, Fiji 2.35, Dynamo1.1.319<br>CryoEM data processing: RELION-4, Coot 0.8.9.2, Phenix 1.17.1, CTFFIND-1.14<br>Mass spectrometry: MassLynx V4.1

For manuscripts utilizing custom algorithms or software that are central to the research but not yet described in published literature, software must be made available to editors and reviewers. We strongly encourage code deposition in a community repository (e.g. GitHub). See the Nature Portfolio guidelines for submitting code & software for further information.

Data

Policy information about availability of data

All manuscripts must include a data availability statement. This statement should provide the following information, where applicable:

- Accession codes, unique identifiers, or web links for publicly available datasets
- A description of any restrictions on data availability
- For clinical datasets or third party data, please ensure that the statement adheres to our policy

The single-particle cryoEM data (refined maps, half-maps and model) of ex vivo AppNL-G-F fibril structure are available in the PDB and EMDB under accession codes

8BFA and EMD-16018 [https://www.ebi.ac.uk/pdbe/entry/emdb/EMD-16018], respectively. The single-particle cryoEM data (refined maps, half-maps and model) of control ex vivo methoxy-X04-treated ex vivo AppNL-G-F fibril structure are available in the PDB and EMDB under accession codes under accession codes 8BFB and EMD-16019 [https://www.ebi.ac.uk/pdbe/entry/emdb/EMD-16019], respectively. All raw cryoEM and tomographic datasets were deposited at EMPIAR. Single-particle data: EMPIAR-11507 [https://www.ebi.ac.uk/empiar/EMPIAR-11507], EMPIAR-11508 [https://www.ebi.ac.uk/empiar/EMPIAR-11508]. In-tissue tomograms: EMPIAR-11509 [https://www.ebi.ac.uk/empiar/EMPIAR-11509].

Human research participants

Policy information about studies involving human research participants and Sex and Gender in Research.

Reporting on sex and gender	Not applicable because this study did not involve human research participants.
Population characteristics	Not applicable because this study did not involve human research participants.
Recruitment	Not applicable because this study did not involve human research participants.
Ethics oversight	Not applicable because this study did not involve human research participants.

Note that full information on the approval of the study protocol must also be provided in the manuscript.

Field-specific reporting

Please select the one below that is the best fit for your research. If you are not sure, read the appropriate sections before making your selection.

☒ Life sciences ☐ Behavioural & social sciences ☐ Ecological, evolutionary & environmental sciences

For a reference copy of the document with all sections, see nature.com/documents/nr-reporting-summary-flat.pdf

Life sciences study design

All studies must disclose on these points even when the disclosure is negative.

Sample size	No sample size calculation was performed because β-amyloid deposition is highly reproducible in 11-14 month old App^NL-G-F mice and was validated by Mx04 cryoCLEM. This was also confirmed by the reproducibility of features observed in all cryoET (see supplementary Table 1) and cryoEM data (see supplementary Fig. 10a). A minimum of 2 and 4 mice were used for in-tissue App^NL-G-F and App^WT/WT-Psd95^GFP/GFP cryoET data, respectively. A minimum of 2 mice were used for cryoEM structure of Arctic Aβ. A minimum of 2 mice were used for cryoEM structure of Arctic Aβ with methoxy-X04.
Data exclusions	No data were excluded from analysis.
Replication	Experiments were replicated at least 3 times. All attempts at replication were successful.
Randomization	Not applicable because cryoET datasets were not in multiple sample groups. For cryoEM structure determination gold standard randomisation was performed by dividing the dataset into two random halves and processing them independently to generate the final map. For all other experiments involving experimental groups animals were separated into groups on the basis of genotype.
Blinding	Not applicable because cryoET data were not separated into sample groups. For annotation of non-amyloid constituents of in-tissue tomograms (described in Extended Data Table 1) was performed blind by two curators. For all other experiments, investigators were blinded to group allocation during data collection and analysis.

Reporting for specific materials, systems and methods

We require information from authors about some types of materials, experimental systems and methods used in many studies. Here, indicate whether each material, system or method listed is relevant to your study. If you are not sure if a list item applies to your research, read the appropriate section before selecting a response.

Materials & experimental systems

n/a	Involved in the study	
☐	☒	Antibodies
☒	☐	Eukaryotic cell lines
☒	☐	Palaeontology and archaeology
☐	☒	Animals and other organisms
☒	☐	Clinical data
☒	☐	Dual use research of concern

Methods

n/a	Involved in the study	
☒	☐	ChIP-seq
☒	☐	Flow cytometry
☒	☐	MRI-based neuroimaging

Antibodies

Antibodies used	6E10: Mouse IgG1 anti-amyloid-beta 1-16/APP (Biolegend, 803001)<br>anti-mouse-IgG1-AF-633 (Lifetechnologies, A21126)
Validation	6E10 primary antibody was validated with control animals (App^WT/WT), see Supplementary Figure 1b.

Animals and other research organisms

Policy information about studies involving animals; ARRIVE guidelines recommended for reporting animal research, and Sex and Gender in Research

Laboratory animals	App^NL-G-F/NL-G-F knockin mice on a c57b/l6 background. Control App^WT/WT - Psd95^GFP/GFP knockin mice on c57b/l6 background.
Wild animals	The study did not involve wild animals.
Reporting on sex	Only male animals were used in the study. Sex based analysis is not necessary because differences in the architecture of amyloid plaques is not expected to be significantly different in the male versus female App.
Field-collected samples	Study did not involve field-collected samples.
Ethics oversight	Oversight and approval was provided by the University of Leeds Animal Welfare and Ethics Review Board.

Note that full information on the approval of the study protocol must also be provided in the manuscript.
