# Supplementary material for: The in-tissue molecular architecture of β-amyloid pathology in the mammalian brain
Source: Nat Commun. 2023 May 17;14:2833. doi: 10.1038/s41467-023-38495-5 (PMC10192217; doi:10.1038/s41467-023-38495-5)
Supplement: Supplementary file 6 [file 41467_2023_38495_MOESM1_ESM.docx]

**Supplementary Information**

**Title:** The in-tissue molecular architecture of amyloid pathology in the mammalian brain. 

Conny Leistner^1^, Martin Wilkinson^2^, Ailidh Burgess^1,3^, Charlie Alexander Lovatt^1^, Stanley Goodbody^1^, Yong Xu^2,3^, Susan Deuchars^1^, Sheena E. Radford*^2^, Neil A. Ranson*^2^, René Frank*^1^

^1^ Astbury Centre for Structural Molecular Biology, School of Biomedical Sciences, Faculty of Biological Sciences, University of Leeds, Leeds, LS2 9JT, United Kingdom.

^2^ Astbury Centre for Structural Molecular Biology, School of Molecular and Cellular Biology, Faculty of Biological Sciences, University of Leeds, Leeds, LS2 9JT, United Kingdom.

^3^ Current Address: Francis Crick Institute, 1 Midland Rd, London NW1 1AT, United Kingdom.

^4^ Current address: Astra Zeneca, Francis Crick Avenue, Cambridge, CB2 0QH, United Kingdom

* Corresponding authors: r.frank@leeds.ac.uk, n.a.ranson@leeds.ac.uk, s.e.radford@leeds.ac.uk

**Inventory of supplementary Tables and Figures**

1. **Supplementary Table 1**. Cryo-EM data collection, refinement and validation statistics for the ex vivo fibril datasets.

2. **Supplementary Figure 1**. Immunohistochemical and cryoFM imaging of methoxy-X04 labelled amyloid plaques in *App*^*NL-G-F*^ mouse brain.

3.  **Supplementary Figure 2.** Quantitative assessment of the in-tissue architecture of amyloid plaques.

4. **Supplementary Figure 3**. Representative tomographic slices from four tomograms. (a-d) collected within central regions of methoxy-X04-labelled amyloid plaques. Supplementary 

5.  **Supplementary Figure 4.** Comparison of extracellular non-amyloid constituents identified tomographic volumes of tissue cryo-sections from *App*^*NL-G-F*^ forebrain MX04-labelled amyloid plaques and *App*^*WT/WT*^ - *Psd95*^*GFP/GFP*^ forebrain lacking amyloid plaques.

6. **Supplementary Figure 5.** Representative tomographic slices from four tomograms (a-d) collected at peripheral regions of methoxy-X04-labelled amyloid plaques.

7. **Supplementary Figure 6.** Tomographic slices related to Figure 4.

8. **Supplementary Figure 7.** Single-particle cryoEM processing scheme for the extracted *App*^*NL-G-F*^ Aβ42 dataset.

9. **Supplementary Figure 8**. Mass spectrometry, solvent-exposed charge and the incompatibility of wt Aβ_1-42 _sequence with the structure of Arctic (E22G) in *ex vivo* *App*^*NL-G-F*^ β-amyloid. 

10.**Supplementary Figure 9**. Cryo-ET of sarkosyl-extracted ex vivo amyloid.

11. **Supplementary Figure 10**. Cryo-EM of amyloid purified from MX04-labelled *App*^*NL-G-F*^ mice and fibril width measurements Supplementary 

12. **Supplementary Figure 11**.  In-tissue and ex vivo cryoET evidence of protofilaments and branched amyloid. 

13. **Supplementary Figure 12**. In-tissue tomogram showing additional examples of branched and unbranched Aβ fibrils in *App*^*NL-G-F*^ amyloid plaques.

**Supplementary Table 1. Cryo-EM data collection, refinement and validation statistics for the ex vivo fibril datasets**

|  | *App*^*NL-G-F*^ Ab_42_ (EMDB-16018) (PDB 8BFA) | *App*^*NL-G-F*^ Ab_42_ (+MX04) (EMDB-16019) (PDB 8BFB) |
| --- | --- | --- |
| **Data collection and processing** |  |  |
| Magnification | 96,000 | 130,000 |
| Voltage (kV) | 300 | 300 |
| Detector | Falcon4 | Falcon4-Selectris |
| Pixel size (Å) | 0.83 | 0.94 |
| Electron exposure (e^–^/Å^2^) | 52 | 41 |
| Exposure rate (e^–^/pixel/s) | 4.5 | 6.1 |
| Nominal defocus range (μm) | -1.6 to -3.1 | -1.4 to -2.9 |
| Movies collected | 2,428 | 4,165 |
| Initial particle images (no.) | 63,680 | 136,214 |
| Final particle images (no.) | 2,568 | 3,640 |
| Symmetry imposed | C1 | C1 |
| Map resolution (Å)     FSC threshold | 3.0 0.143 | 3.2 0.143 |
| Map resolution range (Å) | 3.0-6.9 | 3.1-5.5 |
| Helical parameters     Helical twist (°)     Helical rise (Å)     Crossover (nm) | 179.352 2.418 66 | 179.355 2.414 66 |
|  |  |  |
| **Refinement** |  |  |
| Initial model used (PDB code) | 8BFB | de novo |
| Map sharpening *B* factor (Å^2^) | -23 | -25 |
| Model resolution (Å)     FSC threshold | 2.8 0.143 | 2.9 0.143 |
| Model to map correlation | 0.82 | 0.83 |
| Model composition     Non-hydrogen atoms     Protein residues total     Protein residues modelled     Chains per helical layer     Helical layers modelled | 2860 380 1-38 2 5 | 2860 380 1-38 2 5 |
| *B* factors (Å^2^)     Protein | 89 | 52 |
| R.m.s. deviations     Bond lengths (Å)     Bond angles (°) | 0.002 0.376 | 0.003 0.433 |
| Validation     MolProbity score     Clashscore     Poor rotamers (%) | 1.4 6.5 0.0 | 1.5 9.2 0.0 |
| Ramachandran plot     Favored (%)     Allowed (%)     Disallowed (%) | 100.0 0.0 0.0 | 100.0 0.0 0.0 |

**Supplementary Figure 1**. **Immunohistochemical and cryoFM imaging of methoxy-X04 labelled amyloid plaques in** ***App***^***NL-G-F***^ **mouse brain.** Representative images of experiments that have been repeated independently 3 times with similar results. (**a**) Immunofluorescence confocal microscopy images of amyloid plaques in fixed sections of *App*^*NL-G-F*^ mouse cortex. *Left panels*, Methoxy-X04-labelled plaques pseudo-coloured cyan. *Middle panels*, APP/Aβ detected with 6E10 antibody pseudo-coloured magenta. *Right panels*, overlay of left and middle images. *Bottom middle panel*, shows control sample in which 6E10 primary antibody was omitted. Scale bar, 50 μm. (**b**) Immunofluorescence confocal microscopy images of fixed tissue sections stained with methoxy-X04 from *App*^*NL-G-F*^ and wild-type control mouse cortex, *left* and *right* panels, respectively. Scale bar, 50 μm. (**c**) Cryogenic fluorescence microscopy of high-pressure frozen mouse cortex from *App*^*NL-G-F*^ and wild-type control mice that received i.p. injection of methoxy X04 on *left* and *right*, respectively.  Note the lack of puncta of methoxy X04 in the wild-type mice. Scale bar, 50 μm. 

**Supplementary Figure 2**. **Quantitative assessment of the in-tissue architecture of amyloid plaques.** (**a**) *Left*, Tomographic slice showing region with parallel bundles of amyloid from in-tissue tomograms of methoxy-X04 stained *App*^*NL-G-F*^ amyloid plaque. Yellow box, region analysed by fast Fourier transformation (FFT) shown *Middle*. Scale bar, 20 nm. *Right*, profile of Fourier-transformed image showing reciprocal space distance versus pixel intensity (arbitrary units).   A single peak away from the origin indicate the presence of parallel bundle. The peak indicates inter-fibril distance from the centre of one fibril to the next of amyloid plaques arranged in a parallel bundle.  (**b**) *Left,* Same as **a** but for region showing amyloid not organised in a parallel bundle, indicated by a broad range of spatial frequencies. *Right*, The distance between parallel fibrils measured by FFT analysis as in (a) is plotted for each tomogram.  (**c**) The variability of inter-fibril distances of parallel bundles of fibrils in MX04-labelled β-amyloid plaques (n=2 *App*^*NL-G-F*^ mice). Inter-fibril distance was quantified as described in **a** and plotted for all tomograms with parallel bundles of β-amyloid fibrils**.**  

 **Supplementary Figure 3**. **Representative tomographic slices from four tomograms. (a-d) collected within central regions of methoxy-X04-labelled amyloid plaques**. Scale bar, 100 nm. Filled and open cyan arrowhead, β-amyloid fibril oriented in the x/y plane and along the z-axis of the reconstructed tomogram, respectively. Red arrowhead, spherical exosome. Purple arrowhead, squashed extracellular vesicle. Yellow arrowhead, extracellular droplet. Brown arrowhead, extracellular multilamellar body. Open green arrowhead, plasma membrane of subcellular compartment. Light green arrowhead, ribosome. White arrowhead, localised knife damage in tissue cryo-section. See **Methods** section for criteria used to identify macromolecular and cellular constituents of tomograms. Scale bar, 100 nm

**Supplementary Figure 4. Comparison of extracellular non-amyloid constituents identified in tomographic volumes of tissue cryo-sections from** ***App***^***NL-G-F***^ **forebrain MX04-labelled amyloid plaques and App**^**WT/WT**^ **-** ***Psd95***^***GFP/GFP***^ **forebrain lacking amyloid plaques.** In tissue tomographic slices showing extracellular vesicles from **(a)** *App*^*WT/WT*^ - *Psd95*^*GFP*^ knockin forebrain. Salmon arrowhead, exosome. Scale bar 50 nm. **(b)** *App*^*NL-G-F*^ forebrain MX04-labelled amyloid plaques.  Salmon arrowhead, exosome. Brown arrowhead, multi-lamella body. Purple arrowhead, ellipsoidal vesicle. Orange arrowhead, C-shaped membrane. Scale bar 50 nm.

 **Supplementary Figure 5**. **Representative tomographic slices from four tomograms (a-d) collected at peripheral regions of methoxy-X04-labelled amyloid plaques.** Filled and open cyan arrowhead, β-amyloid fibril oriented in the *x*/*y* plane and along the *z*-axis of the reconstructed tomogram, respectively. Purple arrowhead, mitochondria. Open green arrowhead, plasma membrane of subcellular compartment. Light green arrowhead, ribosome. Pink arrowhead, rough endoplasmic reticulum. White arrowhead, localised knife damage in tissue cryo-section. (**a**) related to Fig. 4 (schematic vii). See **Methods** section for criteria used to identify macromolecular and cellular constituents of tomograms. Scale bar, 100 nm. 

**Supplementary Figure 6**. **Tomographic slices related to Figure 4**. 

(**a**) Cryogenic fluorescence microscopy image of dextran-Alexa fluo-647-labelled tissue cryosections in magenta that mark the extracellular space. Yellow boxes, indicate regions in which tomograms were collected (related to **Fig. 2c)**. Scale bar, 4 μm. (**b-e**) Representative tomographic slices from 5 tomograms corresponding to schematic panels schematics i, iii, iv, v, and vi, respectively. Filled and open cyan arrowhead, β-amyloid fibril oriented in the *x*/*y* plane and along the *z*-axis of the reconstructed tomogram, respectively. Purple arrowhead, mitochondria. Open green arrowhead, plasma membrane of subcellular compartment. Light green arrowhead, ribosome. Red arrowhead, spherical exosome. Brown arrowhead, multilamellar body. White arrowhead, localised knife damage in tissue cryo-section. See **Methods** section for criteria used to identify macromolecular and cellular constituents of tomograms. Scale bar, 100 nm.

**Supplementary Figure 7**. **Single-particle cryoEM processing scheme for the extracted** ***App***^***NL-G-F***^ **Aβ42 dataset.** (**a**) A representative fibril-containing micrograph from the data, with scale bar length of 50 nm. The most populated 2D class averages are shown from the first round of classification of the extracted helical segments. After removing picking artefacts, the data were split into two fibril subsets as highlighted by green or blue boxes. (**b**) Representative 2D class averages from classification of the major fibril subset. Boxed inset shows an initial model for the form after 3D classification of a template generated from 2D class averages of the data. (**c**) Representative 2D class averages from classification of the minor fibril subset. Boxed inset shows an initial model for the form after 3D classification of a template generated from 2D class averages of the data. (**d**) Results of the first round of unbinned 3D classification of the minor fibril form, showing central slices of each output map. Despite further processing, the unambiguous fibril structure could not be determined. (**e**) Results of the first round of unbinned 3D classification showing central slices of each output map which display the same polymorph but with different resolutions. The more ordered class was selected for further processing is highlighted in green. (**f**) Results of the second round of unbinned 3D classification with helical searches, the selected output class is highlighted. (**g**) The refined, sharpened maps before and after CTF refinement and polishing, each coloured by zone based on the two protein chains (as in **Fig. 5c**). (**h**) Local resolution colouring of the final deposited map as calculated by RELION4.

**Supplementary Figure 8**. **Mass spectrometry, solvent-exposed charge and the incompatibility of wt Aβ**_**1-42**_ **sequence with the structure of Arctic (E22G) in** ***ex vivo*** ***App***^***NL-G-F***^ **β-amyloid.** (**a**) Representative mass spectrometric detection of Aβ in sarkosyl-extracted ex vivo amyloid purified from *App*^*NL-G-F*^ forebrain. Parent ions were identified as Aβ_1-42_ E22G, Aβ_1-38_ E22G, Aβ_11-42_ E22G, and Aβ_1-40_ E22G shown in red, blue, yellow, green and purple, respectively. (**b**) Summary table showing, sequence, relative abundance, theoretical molecular weight, observed molecular weight, and charge states of Aβ peptide parent ions in sarkosyl-extracted *ex vivo* amyloid purified from *App*^*NL-G-F*^ forebrain detected by mass spectrometry. (**c**) Atomic models showing solvent-accessible surface coloured by charge of *App*^*NL-G-F*^, Type I, and Type II Aβ fibrils. (d-e) A glutamate at position 22 is not compatible with the *App*^*NL-G-F*^ amyloid fold. (d) Section of the model of the extracted *App*^*NL-G-F*^ fibril structure showing the location of glycine 22 (coloured cyan), with alternating peptide layers within the fibril coloured light and dark grey. (e) Identical to (d) except with the middle Gly22 residue substituted for a glutamate in the model, resulting in major clashes with the proceeding peptide layer (*left*)*.* A perpendicular view of the clash is also shown (*right*). To alleviate such a clash, the peptide backbone would need to significantly rearrange to orient the glutamate side-chain into unoccupied space.

          

**Supplementary Figure 9. Cryo-ET of sarkosyl-extracted ex vivo amyloid.** (**a**) Slice through a representative cryoET reconstruction of sarkosyl-extracted ex vivo amyloid purified from *App*^*NL-G-F*^ forebrain. Cyan arrowheads, amyloid fibril exhibiting crossover points. (Scale bar=10nm). **(b-e)** The stereochemical hand of *App*^*NL-G-F*^ ex vivo Aβ fibrils by cryoET. Tomograms were collected and reconstructed with a rotation angle of +95.5, which has been verified with samples of known chirality to reconstruct tomograms with the correct handedness. To determine whether fibrils were left or right-handed, the direction of protofilaments at the front and back of cross-over points in the atomic model and cryoET data were compared.  **b)** *Left*, schematic showing left-handed helix with protofilaments crossing over at the front (dark blue) from top-right to bottom-left and protofilamants crossing over at the back (light blue) from top-right to bottom left. *Right*, atomic model of App-NL-G-F beta-amyloid reconstructed with left-handed twist.  **c)** *Left* and *right*, show tomographic slice from the front (z-slice 12) of ex vivo purified *App*^*NL-G-F*^ *ex vivo* amyloid and atomic model with clipping planes showing only the front of the amyloid fibril, respectively. Dark blue arrowheads indicate 'front' protofilaments crossing over from top-left to bottom right.  **d)** *Left* and *right*, show tomographic slice from the middle (z-slice 6) of ex vivo purified *App*^*NL-G-F*^ *ex vivo* amyloid and atomic model with clipping planes showing only the middle of the amyloid fibril, respectively. **e)** *Left* and *right*, show tomographic slice from the front (z-slice 1) of ex vivo purified *App*^*NL-G-F*^ *ex vivo* amyloid and atomic model with clipping planes showing the back of the amyloid fibril, respectively. Light blue arrowheads indicate protofilaments crossing over at the back of the fibril axis from top-right to bottom-left. Scale bar, 10 nm

**Supplementary Figure 10**. **Cryo-EM of amyloid purified from MX04-labelled** ***App***^***NL-G-F***^ **mice and fibril width measurements.** (**a**) The deposited cryoEM maps from each *ex vivo* purified amyloid sample, from mice without (*above*) and with MX04 labelling (*below*). The central map slice shows a section corresponding to a single helical layer from each, next to the cryoEM density map coloured by subunit and the FSC curves for the corrected masked map versus the phase randomised maps from postprocessing. (**b**) Angular distribution of the particle sets in the final 3D refinement from *App*^*NL-G-F*^ ex vivo purified amyloid fibrils (*left*) and from MX04-labelled *App*^*NL-G-F*^ mice (*right*) respectively*.*  (**c**) Measurement of fibril widths from the extracted App^NL-G-F^ single-particle cryoEM 2D class averages (*top*) and matched reprojections of the final map after lowpass-filtering to 10 Å (*lower*).

**Supplementary Figure 11.**  **In-tissue and ex vivo cryoET evidence of protofilaments and branched amyloid.** (**a**) Box plot showing distribution of fibril diameters (in nm) measured in 12 in-tissue tomograms of methoxy-X04 labelled amyloid in *App*^*NL-G-F*^ mice (n=2 *App*^*NL-G-F*^ mice; whiskers, min/max; box, Q1 and Q3 quartiles; centre, median). (**b**) Cryogenic fluorescence microscopy images of methoxy-labelled amyloid plaueus *App*^*NL-G-F*^ cryo-sections. Methoxy-X04 fluorescent signal is pseudo-coloured cyan. *Left*, amyloid plaque from which tomogram DS1_TS_06 was collected, highlighted by red box with an average fibril with of 4.73 nm.  *Middle*, amyloid plaque from which tomogram DS2_TS_02 was collected, highlighted by red box with an average fibril width of 6.58 nm. *Right*, amyloid plaque from which tomogram DS2_TS_02 was collected, highlighted by light green box with an average fibril width of 5.48 nm.  (Scale bar=10 μm).(**c-e**) Examples of rare, branched fibrils identified in cryoET data of sarkosyl-extracted ex vivo amyloid purified from *App*^*NL-G-F*^ mice. *Left* panels, tomographic slices of ex vivo amylod cryoET reconstructions. Blue arrowheads, 4-13 nm fibril. Green arrowheads, fibril branch point. Magenta arrowheads, protofilament. Scale bar, 10 nm. *Right* panels, raw tomographic density of branched amyloid.  (**f**) Same as (**c**-**e**) except *middle* panel shows raw tomographic density of branched fibril in magenta and *right* panels shows additional fibrils that contain branch points (in blue, pink, blue and green). Yellow and gold fibrils are single 3-5 nm diameter protofilaments.

**Supplementary Figure 12. In-tissue tomogram showing additional examples of branched and unbranched Aβ fibrils in** ***App***^***NL-G-F***^ **amyloid plaques.** (**a**) *Left*, Tomographic slice of in-tissue amyloid with fibrils oriented on the z-axis of reconstructed tomographic volume. Scale bar, 50 nm. Dashed yellow box indicates close-up shown on *Right*.  Cyan arrowheads, high-contrast spot corresponding to a single fibril. Scale bar, 10 nm.  (**b**) Segmented tomographic density of fibrils shown in (**b**), viewed from the side. Magenta arrowheads, putative branch points. Blue arrowheads, unbranched fibril. (**c**) Representative raw tomographic density of straight, unbranched isolated in-tissue fibrils in Mx04-labelled amyloid plaques. Cyan, atomic model Arctic Aβ fibril fitted into map.
